# The Effects of a Weighted Football Intervention on Ball Velocity of a Standard Football Place-Kick among Elite Gaelic Football Goalkeepers: A Single-Subject Designed Study

**DOI:** 10.3390/sports10110166

**Published:** 2022-10-28

**Authors:** Sam Jermyn, Cian O’Neill, Seán Lacey, Edward K. Coughlan

**Affiliations:** 1 Department of Sport, Leisure & Childhood Studies, Munster Technological University, T12 P928 Cork, Ireland; 2 Research Integrity & Compliance Officer, Munster Technological University, T12 P928 Cork, Ireland; 3 Movement & Skill Acquisition Ireland, T12 P928 Cork, Ireland

**Keywords:** goalkeepers, kickout, overload, skill acquisition, Gaelic football

## Abstract

Weighted football place-kicking acutely enhances the ball velocity (BV) of subsequent standard football place-kicks. However, there is a dearth of research examining the long-term effects of such interventions, with less evidence in existence among elite athlete cohorts. Therefore, the purpose of this study was to investigate the individual effects of a 4 week, eight-session weighted Gaelic football intervention on BV of standard Gaelic football place-kicks among six elite male Gaelic football goalkeepers. This research design was based on a pre-, mid-, post-, and retention-test design. A linear mixed model analysis was employed, with time and participants as fixed effects, and the number of place-kicks per testing session as a random effect. Post hoc tests revealed significant changes in BV for five of the six participants (*p* < 0.05), with three participants experiencing significant BV increases from pre-test to post-test (*p* < 0.05), while no significant differences were found between post-test and retention-test. The remaining three participants experienced no significant BV differences from pre-test to post-test and retention-test. These findings suggest that a weighted football place-kicking intervention can be a time-efficient means of maintaining and enhancing BV and, thus, kick distance, among elite goalkeepers during pre-season and in-season phases.

## 1. Introduction

Weighted implement training (WIT) involves the performance of sport-specific motor skills with heavier-than-normal sporting implements, e.g., baseball bats, cricket balls, and golf clubs [1]. Typically, full range of motion and similar kinematics of the respective motor skill are maintained concurrent to minimal changes in the force–velocity profile of the movement because of the overload applied [2,3,4]. WIT research has focused on the effects of training with bespoke implements on respective sport-specific motor skill performance, primarily in baseball [5] and cricket [6]. Although WIT improves the integral performance variables of the respective motor skill(s) (e.g., ball velocity when pitching a standard 5 oz baseball) [5], there is a paucity of research that has investigated the effects of weighted football training on place-kicking. Similar to baseball and other overhand throwing sports (e.g., American football), whereby improvements in implement velocity and distance are deemed desirable [7], enhancements of ball velocity (BV) and distance are also of great importance in football codes such as Gaelic football, soccer, rugby, and Australian football [8]. Citing the effectiveness of WIT in throwing sports [9], Ball [10] conducted the only known published examination of the effects of a multisession, multiweek weighted football intervention on kicking performance and found that a 4 week combined weighted Australian football (11.1% weight increase) and regulation football training program significantly increased kick distance (*p* < 0.05). Kick distance at post-test was also significantly greater than that of a control group (*p* < 0.05) that demonstrated no significant increases (*p* > 0.05).

**Figure 1:**
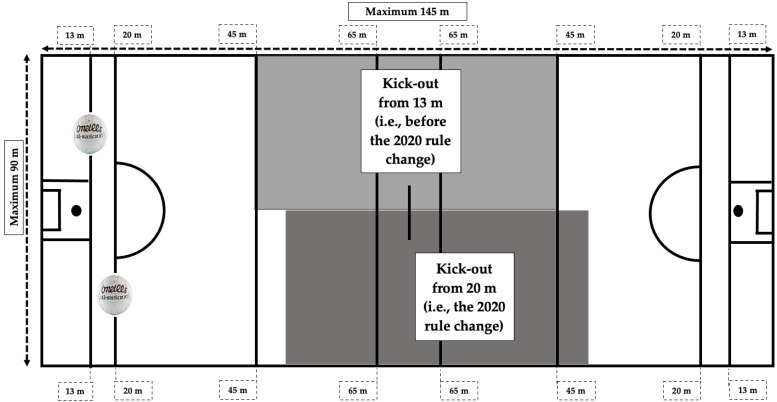
The impact of the 2020 kickout location rule change on the kickout.

Enhanced BV is a major contributor to increased kick distance [11], which is desirable among Gaelic football goalkeepers due to the frequent requirement to pass the ball to teammates downfield to progress a team’s position in as time-efficient a manner as possible [12,13,14,15]. Long kickouts are those that land outside the goalkeeper’s own 45 m line (i.e., a kickout of >32 m) [14]. However, the literature discussed was conducted prior to kickout rule changes that were implemented in 2020, whereby the kickout is now taken from the 20 m line as opposed to the 13 m line at the time of each of the discussed studies (see Figure 1) [16]. Hence, each referenced kickout distance subsequently discussed is relative to the 13 m line.

In 52 elite-level Gaelic football matches, 70.0% ± 20.0% of kickouts were kicked long [14]. It has also been reported that the mean number of kickouts per match is 44.0 (±9.9), and the most common kickout distance (57.0% ± 19.9%) is to the 65 m line or beyond (i.e., >52 m), followed by kickouts to the 45–65 m zone (33.1% ± 16.6%, i.e., 32–52 m in distance) [15]. The introduction of the “mark” rule in 2020 must also be considered as it was implemented to further encourage long kickouts [14]. This rule states that a player is awarded a “mark” by the referee and has the option of taking a free-kick or playing on immediately upon catch of the ball direct from a kickout, on or past the 45 m line nearest the kickout point [13]. The dimensions of a Gaelic football pitch, which measure a maximum of 145 m × 90 m (length × width) [13], representing a 40% increase in length compared to that of a soccer pitch [12], further enhance the need to develop BV. Enhancing kick distance is also emphasized by the fact that a long kickout strategy facilitates a direct approach to score building [15]. Additionally, the aforementioned kickout location rule change places further emphasis on the importance of enhancing BV as kickouts are likely to land closer to the opposition’s goal, thus increasing teams’ chances of scoring directly from kickouts.

In light of these Gaelic football match play demands, recent research has indicated a potential positive effect of the use of weighted Gaelic footballs on place-kicking performance with a standard Gaelic football [4,17]. Jermyn et al. [17] reported that five place-kicks with a 600 g weighted Gaelic football (25% mass increase) resulted in a nominal but practically meaningful 5.4% acute increase in BV of a standard Gaelic football place-kick 8 min after the conclusion of the weighted football place-kicks among a collegiate cohort. Due to the limited sample size (n = 52), the 5.4% increase was statistically insignificant, but Jermyn et al. [17] showed through a power analysis that, with a sample size increase to 18 participants, this meaningful 5.4% increase would have been statistically significant. Additionally, other place-kicks that took place 2, 4, and 6 min after the final weighted Gaelic football place-kick were greater than the mean baseline BV, thus illustrating a post-activation performance enhancement (PAPE) effect, which is the observed effect of a high-intensity conditioning protocol resulting in acute improvements in voluntary muscular contractions [18,19]. This research, therefore, infers an improvement in standard Gaelic football place-kick BV following a 600 g weighted Gaelic football protocol, which has the potential to benefit kickout distance. As a result of the (i) aforementioned match play demands, (ii) results of existing weighted football research [4,10,17], and (iii) implications of improvements in implement velocity and distance inferred from existing WIT research in other sports, investigating the training and retention effects of a weighted Gaelic football training program on place-kicking performance among elite Gaelic football goalkeepers is required.

Although such an investigation is warranted, Wickington and Linthorne [6] reported varied responses to a weighted cricket ball intervention among elite bowlers. These findings support the recommendations of sport scientists to analyze elite athletes individually [20], as the prevalent group analysis techniques mask intraindividual differences in motor performance and learning. This likely implicates the implementation of physical training practices in the applied elite athlete setting [21,22]. A particularly underused, yet recommended, experimental design to assess intraindividual responses to physical preparation interventions is single-subject design, whereby analysis of multiple individuals’ personal responses to an intervention is conducted [21,23,24]. Furthermore, for elite performance research, whereby the recruitment of large sample sizes is challenging and typically impractical, it has been recommended that a single-subject methodology is not only an appropriate and effective approach to examine individual responses to physical training interventions but is indeed recommended over other methodologies due to the need to study and statistically analyze, individual training responses [20,21,23,24,25]. Critically, these study designs and their respective findings are deemed more flexible and practical in applied coaching practices and settings compared to group research designs [21,24]. Therefore, the purpose of this study was to examine the intraindividual effects of a 4 week, eight-session weighted Gaelic football training intervention, comprising a protocol akin to the protocol of Jermyn et al. [17] on the BV of a standard Gaelic football place-kick among elite Gaelic football goalkeepers. It was hypothesized that the intervention would lead to varying levels of BV increases across the testing period for each participant.

## 2. Materials and Methods

This research took place during the COVID-19 pandemic; therefore, the single-subject experimental design was developed in full adherence to local, national, and international health and safety guidelines [26].

### 2.1. Participants

Six elite male Gaelic football goalkeepers participated in this study (mean age = 21.8 ± 3.7 years; body mass = 89.2 ± 11.4 kg; height = 190.1 ± 7.6 cm; elite playing experience = 5.2 ± 3.6 years). The inclusion criteria required participants to be (1) playing at an intercounty (i.e., elite) level, and (2) 18 years of age or older. Goalkeepers were excluded from participating in the study if they were currently dealing with an injury and not medically cleared to partake in regular training activities. The small sample size, therefore, is reflective of the study’s aim to exclusively focus on elite-level goalkeepers. Participants were medically cleared to take part in the study and informed consent was obtained from all participants involved in the study. Furthermore, as this participant cohort comprised elite athletes, unrivalled access to and collaboration with the participants’ lead strength and conditioning coach was secured to define and implement physical preparation and warm up endeavors that were effective and familiar for participants so that the risk of injury was minimized in each session. The study was conducted according to the guidelines of the Declaration of Helsinki and was approved by the Institutional Research Ethics Committee. Descriptive statistics of each participants’ age and fundamental anthropometric scores, as well as a note of their Gaelic football goalkeeping background, are presented in Table 1.

### 2.2. Experimental Setup

**Figure 2:**
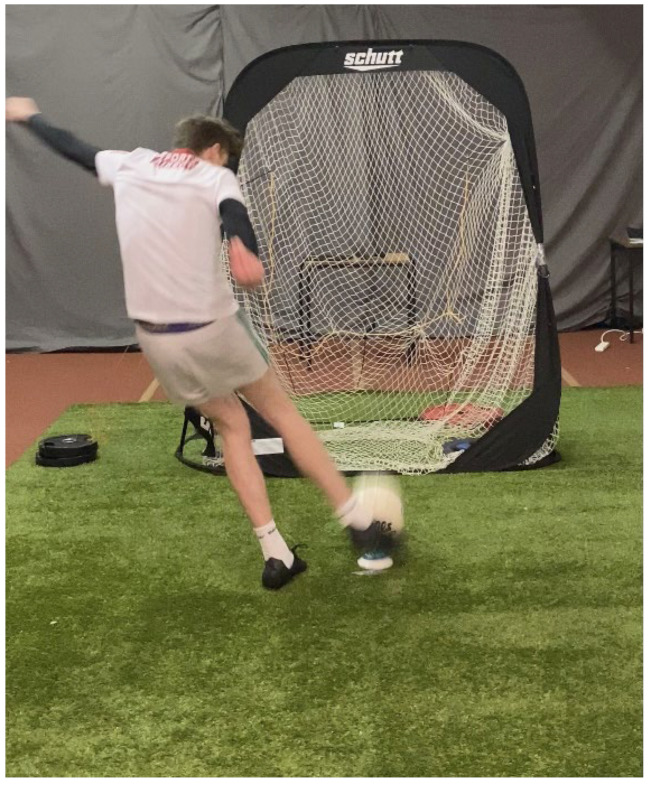
The experimental setup including the training net with rope formation within the net.

The weighted Gaelic football training intervention took place in the host institution’s indoor athletics facility, using a 9.5 m × 3.4 m (length × width) artificial grass playing surface (Synthi Green Sports Surfaces Limited, Co., Cork, Ireland), providing a more representative surface of a football pitch in comparison to the tartan track below, for maximal effort place-kicks to be performed. A Schutt training net (Litchfield, IL, USA), measuring 2.1 m × 1.5 m (height × width), was situated at one end of the artificial grass, thus affording participants the ability to perform a full, unrestricted approach to the ball when performing each place-kick. All place-kicks were executed from a distance of 1.5 m from the center of the net in order to ensure participants were not at risk of making contact with the training net or its frame during the follow-through phase of each place-kick. A 1 m rope was interwoven horizontally into the net at a height of 0.65 m, with additional 1 m ropes interwoven vertically at either end of the horizontal rope. This rope formation served the purpose of guiding ball trajectory toward the center of the net in order to facilitate accurate and reliable measurement of BV, which was attained via a Bushnell radar velocity gun (Bushnell, Overland Park, Kansas). The setting of the radar gun at a height of 1 m and the rope formation ensured reliability and internal consistency of BV measurement. The radar gun was situated behind the goal at a distance of 2.75 m from the front of the training net to ensure a ball would not contact the radar gun as it was kicked into the net. Two RS PRO Halogen Work Light systems (400 W, 220–240 V; Radionics Ltd. Glenview Industrial Estate, Herberton Road, Rialto, Dublin 12, Ireland) were placed to the rear of the synthetic playing surface and angled upward to the ceiling to add greater light to the indoor facility. The experimental setup is displayed in Figure 2.

**Figure 3:**
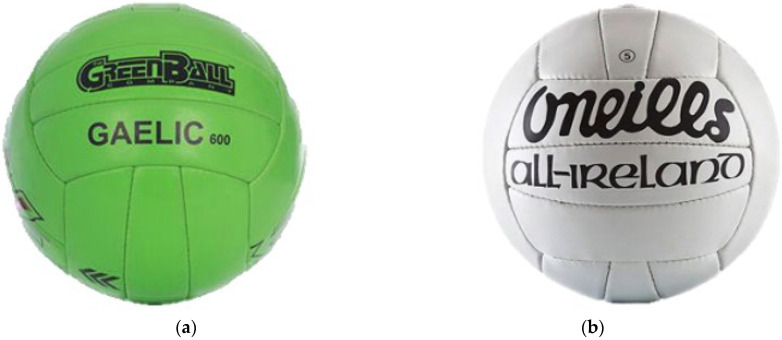
The 600 g weighted Gaelic football (**a**) and 480 g standard Gaelic football (**b**).

The weighted Gaelic football (The Green Ball Co., Dublin, Ireland; Figure 3a) used in this study weighed 600 g, a 25% increase from the standard 480 g game ball. The size and outer paneling of the weighted football was identical to that of a standard Gaelic football, with the additional weight due to a heavier inner bladder. The standard football used in this study was a size 5 O’Neill’s All-Ireland Gaelic football (O’Neill’s Irish International Sports Co. Ltd., Belfast, Northern Ireland; Figure 3b). The standard football was inflated to a pressure of 9.75–10 pound per square inch (PSI), resulting in the recommended mass of 480 g [13].

### 2.3. Procedure

#### 2.3.1. Testing Session Procedure

Upon arrival to the facility for each testing session, participants completed a 10 min warm up comprising of mobility, activation, and dynamic movement phases. While participants were encouraged to complete this warm-up protocol, additional time was allocated so that they could include their own warm up and preparatory endeavors to facilitate maximal performance output in terms of their subsequent place-kicks. This warm-up protocol was developed in consultation with the players’ lead strength and conditioning coach to provide a familiar and effective warm up for all goalkeepers participating in this study.

Upon conclusion of the warm-up phase, the testing session began. In all testing sessions (pre-, mid-, post-, and retention-test), participants performed five maximal effort place-kicks with a standard Gaelic football. Prior to the five test trials, participants performed two familiarization trials with the standard Gaelic football. A minimum inter-trial time interval of 30 s was implemented. To respect the in-game kickout “routine” of each participant, a predetermined maximum inter-trial time interval was not imposed. Across all testing sessions, the minimum inter-trial time interval per individual was 52 s, with the maximum inter-trial time interval per individual being 76 s. Participants were instructed to (1) “kick with maximal effort”, and (2) “kick within the rope formation”. Prior to each trial, participants received the following verbal instruction: “This is your (trial number) maximal effort place-kick. You may proceed when ready”. Participants were permitted to approach all place-kicks as they would in a match situation (i.e., there were no constraints on approach angle or number of approach steps).

Upon conclusion of the pre-test, participants took part in an eight-session training intervention, with the mid-test taking place between the fourth and fifth intervention sessions. A post-test took place within 7 days of the conclusion of the intervention, with a retention-test taking place 7–14 days after the participant’s respective post-test. The procedures of the mid-test, post-test, and retention-test were identical to the pre-test.

#### 2.3.2. Intervention Session Procedure

During intervention sessions, participants completed an identical warm up to that of the testing sessions, followed by two familiarization place-kicks with the weighted football, prior to performing five maximal effort place-kicks with the weighted Gaelic football. A 1 min recovery period was situated between each weighted football place-kick. This was followed by a single place-kick with a standard Gaelic football approximately 2, 4, 6, and 8 min after the final weighted Gaelic football place-kick [17]. For each place-kick with the standard Gaelic football (the phase following the weighted Gaelic football place-kicks), participants were informed that they were permitted to commence the next trial 15 s prior to the respective timepoint. For example, for the standard Gaelic football place-kick at the 2 min timepoint, participants were informed that they could commence the trial 1 min 45 s after the final weighted Gaelic football place-kick.

#### 2.3.3. Season Schedules and Associated Strength and Conditioning Programs

Concurrent to partaking in the weighted Gaelic football training intervention, participants continued their weekly on-field practice and match schedules, as well as associated strength and conditioning programs. Upon commencement of the study, participants were beginning Phase 2 of their pre-season, with Phase 1 concluding 1 week prior. Phase 1 was defined as the phase in which participants were not permitted to perform on-field collective activities as per COVID-19 restrictions. Participants were, however, completing at-home strength and conditioning sessions. Phase 2, therefore, was defined as the phase in which on-field collective activities recommenced. Across the experimental period, all goalkeepers participated in a minimum of two matches.

Due to the COVID-19 restrictions that were in place at the time of both phases of the participants’ pre-season, collective strength training sessions were not permitted. Therefore, across both phases, intensity was prescribed at 60% to 90% of one repetition-maximum to cater to the varied and oftentimes limited equipment and loads that each player had access to. Although loads varied, strength training session structures remained constant across all players and both pre-season phases. The structure of participants’ strength and conditioning and on-field practice schedules at the time of the study (Phase 2) and immediately prior to the study (Phase 1) are detailed in Table 2.

### 2.4. Statistical Analysis

To assess the measurement accuracy of the radar gun, the level of agreement of BV measures between the radar gun used in the study and a Bushnell Speedster III (Bushnell, Overland Park, Kansas) was assessed via the intraclass correlation coefficient (ICC). To assess the test–retest reliability of the radar gun used in this study, the level of agreement of the radar gun’s measurement of BV on three separate occasions was assessed via ICC. Each session consisted of 40 trials whereby a custom-built football projection machine, set at a speed of 1000 rpm, projected a standard size-5 O’Neills Gaelic football toward the speed guns. A customized black cover, with a 50 cm slit down the center, was placed directly in front of the projection machine in order to occlude the spinning wheels from the radar guns to ensure the displayed BV value was that of the oncoming football. For both reliability analyses, ICC estimates and their 95% confidence intervals were calculated on the basis of absolute agreement and a two-way mixed-effects model. For inter-device reliability, average measures results are reported. For intra-device reliability, single measures results are reported. The design of the reliability study and the subsequent reporting of its results are based on the recommendations of Koo and Li [27].

A linear mixed model analysis was carried out to examine the intraindividual effects of the 4 week, eight-session weighted Gaelic football training intervention on BV of standard Gaelic football place-kicks. Time and participants served as fixed effects, while the number of place-kicks per testing session served as a random effect. In order to control for the number of participants in the single-subject design, Tukey Honestly Significant Difference (HSD) post hoc analysis was performed on the participant variable in order to assess intraparticipant changes in BV across the testing period. The model assumptions of distribution normality and homoscedasticity were assessed via Shapiro–Wilk and Breusch–Pagan tests, respectively. All statistical test results were interpreted using a 5% level of significance. Partial eta squared (η^2^) was used to assess the effect size of the linear mixed model (small: 0.01 ≤ η^2^ < 0.06; medium: 0.06 ≤ η^2^ < 0.14; large: η^2^ ≥ 0.14) [28]. Statistical analysis was performed using R Statistical Software (version 4.0.4; R Foundation for Statistical Computing, Vienna, Austria).

## 3. Results

Results revealed that the inter-device reliability was 0.88 [95% CI, 0.75–0.94], indicating good to excellent reliability between the two devices. Intra-device test–retest reliability was 0.85 [95% CI, 0.75–0.92], indicating good to excellent BV measurement reliability.

A linear mixed model analysis was conducted to investigate the intraindividual effects of the 4 week, eight-session weighted Gaelic football training intervention on BV of standard Gaelic football place-kicks. The resulting model satisfied the assumptions of residuals being normally distributed and homoscedastic, as assessed by the Shapiro–Wilk (*p* > 0.05) and Breusch–Pagan (*p* > 0.05) tests, respectively. Results revealed a large, significant interaction effect between participant and time (*F*(15, 96) = 10.85, *p* < 0.0001, partial η^2^ = 0.63). Post hoc analysis examining the participant variable revealed that, with the exception of Participant 3, all other participants (n = 5) experienced significant changes in BV across the testing period (*p* < 0.05). Mean BV values per testing period per participant are presented in Table 3.

**Figure 4:**
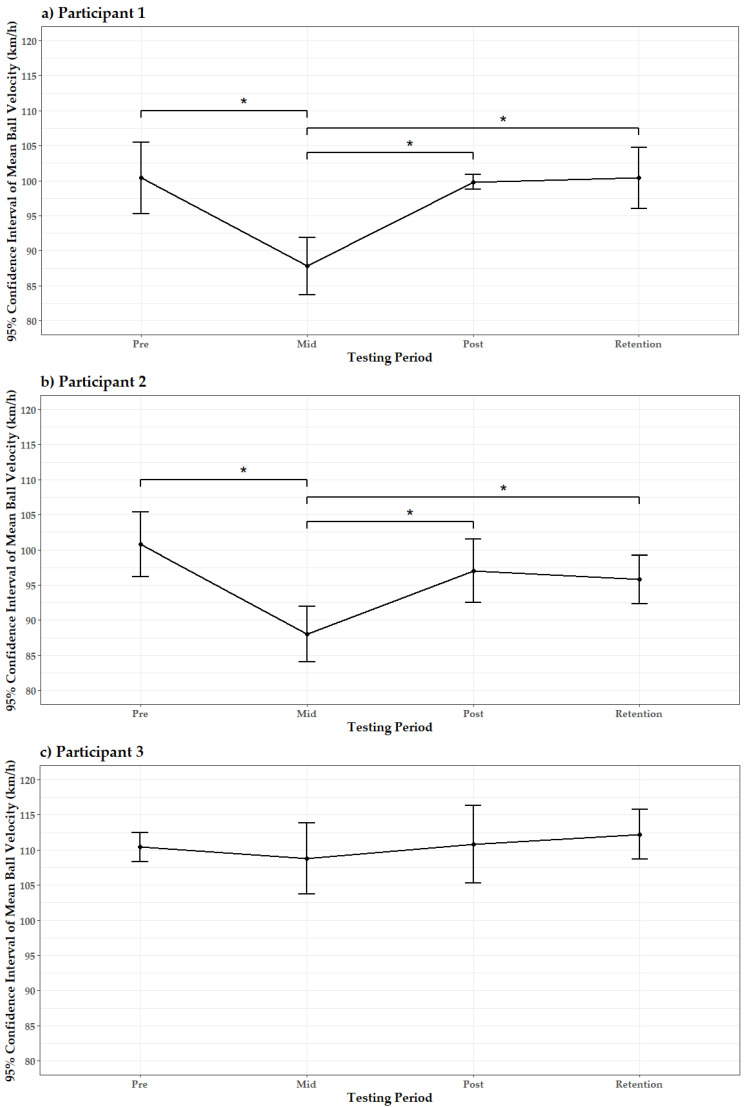

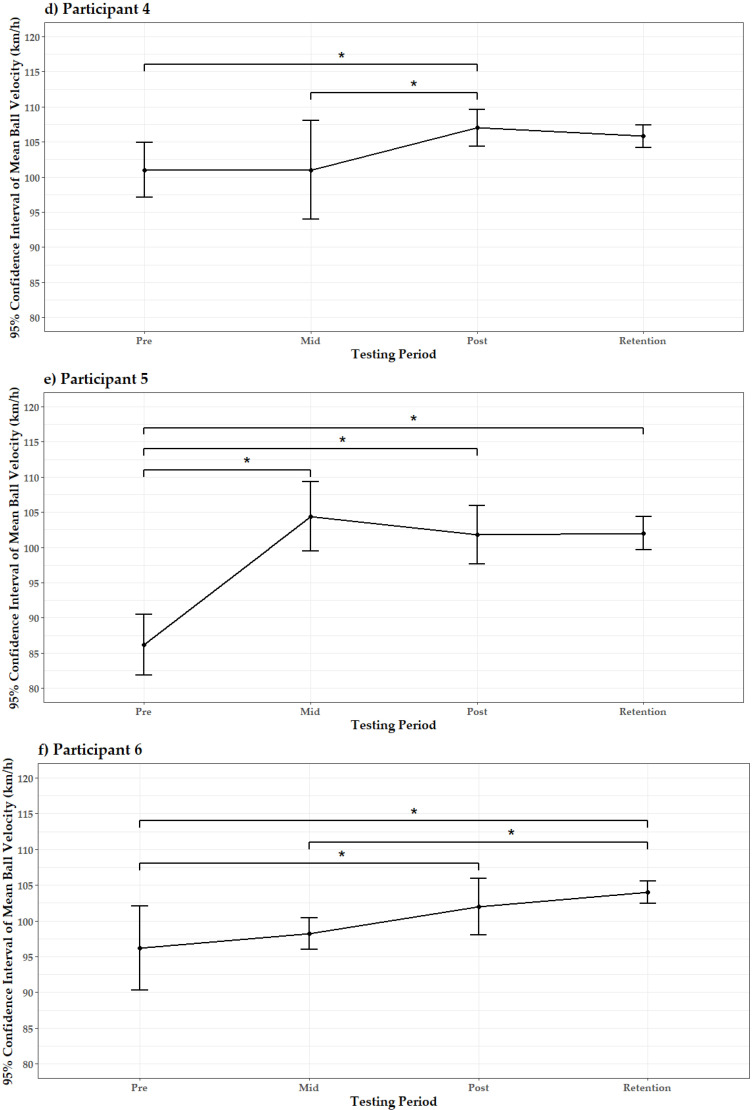
(**a**–**f**) The 95% confidence interval of mean BV across the testing period per participant. An asterisk (*) and corresponding horizontal bracket indicate a statistically significant difference in BV between the respective testing periods (*p* < 0.05).

Graphical representations of mean BV per testing period per participant are displayed in Figure 4a–f. Following the pre-test, Participant 1 experienced a significant decrease in BV at mid-test (*p* < 0.0001). However, BV significantly increased at post-test (*p* < 0.0001) and retention-test (*p* < 0.0001) relative to the mid-test, indicating that BV returned to pre-test magnitudes as there were no significant differences in BV between pre-, post-, and retention-tests (*p* > 0.05) (Figure 4a). Similarly, Participant 2 experienced a significant decrease in mean BV from pre-test to mid-test (*p* < 0.0001), followed by a subsequent significant increase at post-test (*p* = 0.0002) and retention-test (*p* = 0.0018) relative to the mid-test (Figure 4b). Participant 4 experienced a significant increase in BV from pre-test to post-test (*p* = 0.0249) and from mid-test to post-test (*p* = 0.0249). These improvements in BV were maintained through the retention-test as no significant differences were observed between post- and retention-tests (*p* > 0.05) (Figure 4d). Participant 5 experienced a significant increase in BV from pre-test to mid-test (*p* < 0.0001), post-test (*p* < 0.0001), and retention-test (*p* < 0.0001). Participant 5 experienced no other significant differences in BV across the testing period (Figure 4e). Participant 6 experienced a significant increase from pre-test to post-test (*p* = 0.0323) and retention-test (*p* = 0.0018), along with an additional significant increase from mid-test to retention-test (*p* = 0.0323) (Figure 4f). Although the aforementioned five participants experienced significant changes in BV across the testing period (*p* < 0.05), Participant 3 did not experience significant changes in BV. However, a statistically insignificant (*p* > 0.05) mean BV increase of 1.8 km/h was observed across the testing period for this participant (Figure 4c).

## 4. Discussion

Research suggests that WIT results in significant increases in standard implement velocity and distance [5,6,10,29,30]. The majority of this research evaluated the effects of weighted baseball equipment on the sport’s respective motor skills (i.e., batting and pitching). Although a single published study directly assessed the effects of a 4 week, eight-session weighted Australian football program on place-kicking performance, there has been no research into the impact of weighted Gaelic footballs on place-kicking performance of elite Gaelic football goalkeepers. Therefore, due to (i) the findings of previous studies that demonstrated that WIT induces increases in standard implement velocity and distance, (ii) the concurrent interest for improved BV among Gaelic football goalkeepers as enhanced BV majorly contributes to increased kick distance [11], and (iii) the results of previous studies that indicate positive acute effects of a weighted Gaelic football protocol on standard football place-kick performance [4,17], an investigation into the effects of a 4 week, eight-session weighted Gaelic football place-kicking intervention on BV of standard Gaelic football place-kicks among elite goalkeepers was warranted. However, as a response to previous WIT research that highlighted varied intraindividual responses to WIT among elite athletes [6] and the inherent difficulties in recruiting large samples of elite athletes, a multiple participant baseline single-subject design was applied. This experimental design was deemed more suitable for the study of elite athletes compared to the more prominent group analyses techniques [20,24].

The current study found that, in conjunction with participants’ return to weekly on-field team practice and strength and conditioning programs, five of the six participants experienced significant increases and/or decreases in BV across the testing period (*p* < 0.05). Participant 1 and Participant 2 experienced the only significant decreases in BV across the testing period. Although Participant 1 did not report any injury or health-related issues prior to or during the current study, Participant 2 experienced a mild groin strain between the pre-test and the first intervention session. This injury was not sustained because of the testing and intervention sessions. Although the participant was medically cleared to return to all football activities, the significant decrease in BV from pre-test to mid-test suggests this injury may have negatively impacted place-kicking performance. However, as BV significantly increased from mid-test to post-test and retention-test, with no significant differences observed between pre-test, post-test, and retention-test, it is suggested that this training intervention may have supported his return to pre-injury performance levels. Participants 4, 5, and 6 all experienced significant increases in BV from pre-test to post-test. Although Participant 4 did not maintain significant differences in BV from pre-test to retention-test, retention-test BV was still greater than pre-test and mid-test measures, with no significant differences evident between post- and retention-test. In contrast, Participant 5 and Participant 6 experienced significant BV improvements at post-test and retention-test relative to pre-test scores, thus indicating that significant BV improvements incurred throughout the intervention period were retained. In particular, Participant 5 experienced the greatest BV increases among all participants, whereby, relative to pre-test, an 18.2 km/h (21.11% increase) improvement was observed at mid-test, a 15.6 km/h (18.1% increase) improvement was observed at post-test, and a 15.8 km/h (18.33% increase) improvement was observed at retention-test. However, caution is warranted when considering these substantial BV increases as the participant was dealing with an ankle injury just prior to commencement of the study. Although he was medically cleared to participate, any lingering effects of the injury may have impacted upon his performance in the pre-test, thus leading to significant and likely inflated increases in BV in each of the other testing periods. Furthermore, the participant stated, post data analysis, that place-kicking on an artificial surface may have resulted in him performing submaximal effort place-kicks in the initial testing session, although all participants were prompted to kick with maximal effort prior to each experimental and intervention place-kick. Indeed, previous WIT studies have emphasized that the use of artificial performance environments in experimental sessions (i.e., the current study’s use of the artificial playing surface), as well as the use of a net versus representative targets (i.e., teammates), may inhibit elite athletic performance [31]. However, as the purpose of the current study was to investigate BV effects, these experimental settings were required.

Participant 3 was the only participant who did not experience significant changes in BV across the study’s duration. Analysis of mean BV values per participant across testing phases revealed that this participant displayed the greatest mean BV values in each testing period compared to all other participants. Furthermore, this participant displayed the greatest mean BV value across all experimental trials (110.55 km/h), with a maximum value of 117 km/h. Observation of these results may indicate a ceiling effect. Similar results have been observed in studies that have incorporated the use of weighted golf clubs in training interventions, whereby golfers who had already established very high swing speeds experienced no significant improvements in swing speed post intervention, thus illustrating a ceiling effect [32]. Research that investigated the effects of weighted baseballs on pitching performance, however, inferred the need to identify velocity values at which a ceiling effect appears to occur, in order to further refine the practical applications and programming recommendations of WIT [33,34]. Although Sands et al. [20] stated that readers should be aware of the poor generalizability of single-subject analyses to other athletes and cohorts, it is suggested that findings of such study designs may be applicable to players that share similar characteristics to the single-subject study’s participants [25]. On the basis of Participant 3′s results, it may, therefore, be suggested that goalkeepers who display mean BV values of 110 km/h may not experience significant BV increases as a result of implementing a weighted football intervention akin to the program utilized in the current study. Nonetheless, as per the conclusions of Álvarez et al. [32], it is important to note that WIT may facilitate the maintenance of previously achieved velocity gains among the most powerful and explosive athletes. Furthermore, albeit statistically insignificant, Participant 3′s BV values at post-test and retention-test were greater than pre-test values. As evidenced by Participant 3, Participant 6 also experienced a slight BV increase from post-test to retention-test. As participants were not exposed to the weighted football between the post-test and retention-test, it is suggested that these slight increases may have been the result of the participants’ on-field sessions, whereby a respective amount of time per session was allocated to kickout distance.

### Limitations

The authors acknowledge limitations in this study. Firstly, although the purpose of this research was to analyze individual responses to the weighted football intervention, an untreated or standard football control phase per individual was not included. This would have facilitated a determination of whether the weighted football intervention was unique in its ability to evoke the observed performance improvements among some of the participants. Furthermore, in single-subject research designs, a baseline testing period should reflect a period (potentially multiple testing sessions as opposed to the utilized single session pre-test) whereby implementation of the intervention only occurs once per performance across baseline sessions [23]. This, therefore, likely minimizes the contribution of external factors to baseline performance (the contribution of which may be higher if only a single baseline session is conducted) so that a more accurate comparison can be made between post-intervention performance and true baseline performance [35]. However, corresponding to the literature’s acknowledgement of the challenges presented in applied settings when endeavoring to implement chronic baseline phases [35], increased time constraints in the current study resulting from each participant’s forthcoming competitive season schedule meant that extending the study’s duration, to incorporate a control phase and/or an extended baseline phase, was not feasible. To combat this, Kinugasa et al. [21] stated that multiple baseline designs with multiple participants, as per the design of the current study, represent an effective study design to control threats to internal validity. Nonetheless, with respect to these limitations, it is recommended that future research, if possible, repeats this study with an elite athlete cohort but includes an extended baseline testing period, whereby the weighted football intervention is only implemented upon stabilization of baseline BV values.

The small sample size may also be deemed a limitation of the current study. However, as the aim of this research was to investigate the intraindividual effects of a weighted Gaelic football training intervention on place-kicking performance among “elite-level” Gaelic football goalkeepers, and the corresponding limited number of such athletes that meet this criterion, it was not possible to obtain a larger sample size. However, efforts were made to control for this limitation, including collaboration with an expert statistician for the purposes of ensuring use of appropriate data analysis techniques as per the recommendations of Skorski and Hecksteden [25] and Hecksteden, Kellner, and Donath [36].

An additional limitation of this study is the likely variance in ball trajectories and its subsequent impact on accuracy of BV measurement with the radar gun. Although the manufacturers of the radar gun state that the radar gun has a measurement accuracy margin of error of ±2.0 km/h, deviations in ball trajectory likely impacted measurement accuracy due to the relatively fixed direction in which the radar gun was pointed. However, the manufacturers state that an angle of incidence of ≤12° between object trajectory and the radar gun does not affect measurement accuracy, but a deviation of 20° can result in a measurement accuracy margin of error of ±3.0 km/h. While the authors attempted to control for this by requiring participants to aim their kick within the rope formation embedded in the net so that ball trajectories closely aligned with the positioning of the radar gun, it is suggested that additional accuracy constraints to control for trajectory deviations would have negatively affected participants’ intentions to place-kick with maximal effort. Nonetheless, a reliability study showed that inter-device (ICC = 0.88, 95% CI = 0.75–0.94) and intra-device (ICC = 0.85, 95% CI = 0.75–0.92) test–retest reliability was good to excellent. Furthermore, in addition to the researcher manually operating the radar gun in order to align the trajectory of the radar gun toward the kicking location for each trial, the two familiarization trials supported the researcher’s attunement to each participant’s typical kick execution and trajectory, thus further refining the positioning of the radar gun with respect to the trajectory of the ball. Lastly, similar to limitations of other WIT studies, where interventions were implemented at the beginning of the respective athlete cohort’s season [37], the current study was initiated immediately following a COVID-19 “lockdown” period, whereby participants were returning to on-field practice and technical coaching for the first time in 14 weeks. The influence of these on-field team sessions on improvements in BV cannot, therefore, be excluded. However, from a contrasting perspective, the implementation of a weighted football intervention would most likely never occur in isolation in the applied setting as such an intervention would most likely be implemented concurrent to or as part of a strength and conditioning program and regular on-field practice. Therefore, the context within which the current intervention was implemented (i.e., in the middle of a high-performance pre-season) provides practitioners with authentic and meaningful information relating to the interactions between this intervention, concurrent training activities, and the subsequent effects on place-kicking performance.

## 5. Conclusions

The findings of the current study illustrate that a 4 week, eight-session weighted Gaelic football training intervention, comprising a protocol akin to Jermyn et al. [17], may result in significant increases in BV of a standard Gaelic football place-kick among elite level Gaelic football goalkeepers. Improvements in BV may facilitate kickouts of greater distance, a performance outcome deemed desirable in Gaelic football due to (i) the prevalence of long kickouts (>32 m) in match play, (ii) the expansive playing surface, (iii) the recent introduction of the “mark” rule to further encourage long kickouts, and (iv) the potential to create scoring opportunities from long kickouts. However, as per the results of the current study, varied individual responses to the weighted football intervention may be observed, particularly as the individual who displayed the greatest baseline BV in the current study (i.e., Participant 3) experienced no significant BV changes across the experiment.

## 6. Practical Applications

As the utilized program resulted in intervention sessions being completed within 25 min, this intervention provides practitioners, such as skill acquisition specialists and strength and conditioning coaches, with a time-efficient means of inducing intraindividual increases in standard football velocity among elite goalkeepers that can, therefore, be implemented as part of their training programs.

## Figures and Tables

**Table 1:** Participants’ anthropometric profiles and goalkeeping experience.

Participant	Age (Years)	Mass (kg)	Height (cm)	Gaelic Football Goalkeeping Background
1	26	95.6	188.2	13 and 9 years of GK and elite GK experience, respectively
2	26	90.4	185.2	12 and 9 years of GK and elite GK experience, respectively
3	23	102.4	196.5	8 and 5 years of GK and elite GK experience, respectively
4	19	95.7	198.9	13 and 1 years of GK and elite GK experience, respectively
5	18	78.1	178.6	9 and 6 years of GK and elite GK experience, respectively
6	19	72.8	193.3	4 and 1 years of GK and elite GK experience, respectively

Key: GK = goalkeeping.

**Table 2:** Participants’ training activities prior to and during the current study.

On-Field Practice and S&C Endeavors at the Time of the Study (i.e., Pre-Season Phase 2)				
Training Mode	**On-Field Collective Practice**	Kickout Practice	Strength Training	Conditioning
**Frequency**	2 sessions per week	2 sessions per week	2-sessions per week	1 session per week (if not participating in a match)
**Intensity**	Session-dependent	Session-dependent	60–90% 1 repetition-maximum	Varied intensity; session-dependent
**Time**	60 to 90 min	10 to 20 min	60 min	30 to 45 min
**Type**	Collective team training	Various types of kickouts (10–20); varied direction and distance	Upper- and lower-body bilateral and unilateral strength and power exercises	Aerobic and anaerobic running; “soft-skill” activities focused on development of passing, soloing, and first touch
**S&C Endeavors Completed in the Phase Prior to the Study (i.e., Pre-Season Phase-1)**				
**Training Mode**	**On-Field Collective Practice**	**Kickout Practice**	**Strength Training**	**Conditioning**
**Frequency**	N/A	N/A	2 sessions per week	1 to 3 sessions per week
**Intensity**	N/A	N/A	60–90% 1 repetition-maximum	Varied intensity; session-dependent
**Time**	N/A	N/A	60 min	30 to 45 min
**Type**	N/A	N/A	Upper- and lower-body bilateral and unilateral strength and power exercises	Aerobic and anaerobic running; speed and agility development; “soft-skill” activities focused on development of passing, soloing, and first touch

Key: N/A = not applicable; S&C = strength and conditioning.

**Table 3:** Mean ball velocity (BV) (km/h) per testing period per participant.

Participant	Pre-Test Mean ± SD BV (km/h)	Mid-Test Mean ± SD BV (km/h)	Post-Test Mean ± SD BV (km/h)	Retention-Test Mean ± SD BV (km/h)
1	100.4 ± 4.10	87.8 ± 3.27	99.8 ± 0.84	100.4 ± 3.51
2	100.8 ± 3.70	88.0 ± 3.16	97.0 ± 3.61	95.8 ± 2.77
3	110.4 ± 1.67	108.8 ± 4.09	110.8 ± 4.44	112.2 ± 2.86
4	101.0 ± 3.16	101.0 ± 5.66	107.0 ± 2.12	105.8 ± 1.30
5	86.2 ± 3.49	104.4 ± 3.97	101.8 ± 3.35	102.0 ± 1.87
6	96.2 ± 4.71	98.2 ± 1.79	102.0 ± 3.16	104.0 ± 1.22

## Data Availability

The data presented in this study are available from the corresponding author upon request.
